# Inhibition of PDGF-B Induction and Cell Growth by Syndecan-1 Involves the Ubiquitin and SUMO-1 Ligase, Topors

**DOI:** 10.1371/journal.pone.0043701

**Published:** 2012-08-17

**Authors:** Kathleen R. Braun, Allison M. DeWispelaere, Steven L. Bressler, Nozomi Fukai, Richard D. Kenagy, Lihua Chen, Alexander W. Clowes, Michael G. Kinsella

**Affiliations:** 1 Benaroya Research Institute at Virginia Mason, Seattle, Washington, United States of America; 2 Department of Surgery, University of Washington, Seattle, Washington, United States of America; Ottawa Hospital Research Institute, Canada

## Abstract

Syndecans are receptors for soluble ligands, including heparin-binding growth factors, and matrix proteins. However, intracellular targets of syndecan-1 (Sdc-1)-mediated signaling are not fully understood. A yeast two-hybrid protein interaction screening of a mouse embryo library identified the ubiquitin and SUMO-1 E3 ligase, Topors, as a novel ligand of the Sdc-1 cytoplasmic domain (S1CD), a finding confirmed by ligand blotting and co-precipitation with Sdc-1 from cell lysates. Deletion mutagenesis identified an 18-amino acid sequence of Topors required for the interaction with the S1CD. By immunohistochemistry, Topors and Sdc-1 co-localized near the cell periphery in normal murine mammary gland (NMuMG) cells *in vitro* and in mouse embryonic epithelia *in vivo*. Finally, siRNA-mediated knockdown of Topors demonstrated that Topors is a growth promoter for murine arterial smooth muscle cells and is required for the inhibitory effect of Sdc-1 on cell growth and platelet-derived growth factor-B induction. These data suggest a novel mechanism for the inhibitory effects of Sdc-1 on cell growth that involves the interaction between the cytoplasmic domain of Sdc-1 and the SUMO-1 E3 ligase, Topors.

## Introduction

The syndecans are a family of transmembrane heparan sulfate proteoglycans (in mammals, Sdc-1–4) [1], [2]. The short cytoplasmic domains of the different Sdc family members are highly conserved between species and have common structures, with constant regions proximal (C1) and distal (C2) to the transmembrane region, and an intervening variable (V) region. Considerable work has described Sdc structures, developmental expression, and differential localization to cells within different tissues. In addition, a number of elegant studies have firmly established functional roles for Sdcs as receptors or co-receptors for matrix proteins and heparan-binding enzymes, growth factors and cytokines (3,4). Structure-function studies have shown that ligands bind both the protein core and the heparan sulfate chains of Sdc extracellular domains [3]. However, in spite of evidence that some Sdc signaling functions may be mediated by the cytoplasmic domain [4], [5], [6], [7], the mechanism and cellular effects of putative Sdc-1 signal transduction remain unclear.

Immunocytochemical evidence indicates that Sdc-1 preferentially localizes to the basolateral surfaces of epithelial cells and that association with cytoskeletal elements [8] requires the Sdc-1 cytoplasmic domain (S1CD) [9], [10], [11]. Unlike focal adhesion-associated Sdc-4 [12], [13], binding of matrix protein ligands by cells that ectopically express Sdc-1 activates lamellipodial spreading, fascin and actin bundling, and localization of Sdc-1 to fascin-containing cellular spikes in a manner that requires the variable region of the S1CD [14]. Clustering of Sdc-1 in lipid-rich membrane rafts after binding of lipoprotein lipase [15], [16] also requires the S1CD [15]. Several PDZ protein interaction domain-containing adapter proteins isolated using the yeast two-hybrid assay with Sdc-1 bait sequences [17], [18], [19], [20] interact with conserved regions of Sdc-1–4 cytoplasmic domains and mediate interactions with the cytoskeleton, cytoskeleton-associated proteins and some cellular receptors [3]. An additional level of regulation is indicated by the observation that Src and the small GTPase, Rab5, have been shown to bind the S1CD [6], [21]. In addition, the S1CD includes conserved tyrosine residues that can be phosphorylated [22], [23], suggesting that tyrosine kinases and associated phosphorylases are likely to interact with the S1CD. Some of these associations may be transient or ligand-dependent, such as the agonist-induced dissociation of Rab5 from the S1CD that regulates Sdc-1 ectodomain shedding [21]. Thus, the Sdc-1 cytoplasmic domain is clearly required for interactions that are necessary for specific Sdc-1 functions.

In this study, we have screened a cDNA library for S1CD-interactive proteins whose activity may shed light on putative Sdc-1 signaling functions. We have identified Topors as a novel S1CD-interacting protein that co-localizes with Sdc-1 within the peripheral cytoplasm of epithelial cells and co-precipitates with Sdc-1 from cell lysates. Topors is a ubiquitin and SUMO-1 E3 ligase [24], [25] with several previously identified targets [25], [26], [27], [28], [29], [30], [31]. Topors mutations are associated with retinitis pigmentosum [32], [33]. Moreover, Topors is deficient in colon adenocarcinomas and several carcinoma cell lines [24], suggesting that Topors is a tumor suppressor that may function in part by increasing the activity of the tumor suppressor, p53 [34], [35]. However, Topors may also inactivate p53, suggesting the function of Topors may be cell- or context-dependent, which is supported by the results reported here, in which Topors is shown to promote arterial smooth muscle cell (SMC) growth. These studies also provide a potential mechanism for the previously reported growth suppressor activity of Sdc-1 for SMCs [36] and possibly tumors [37], [38] that involves the interaction of Sdc-1 with Topors.

## Results

### LexA-S1CD fusion protein interacts with a novel protein in a yeast two-hybrid protein interaction assay

**Figure 1:**
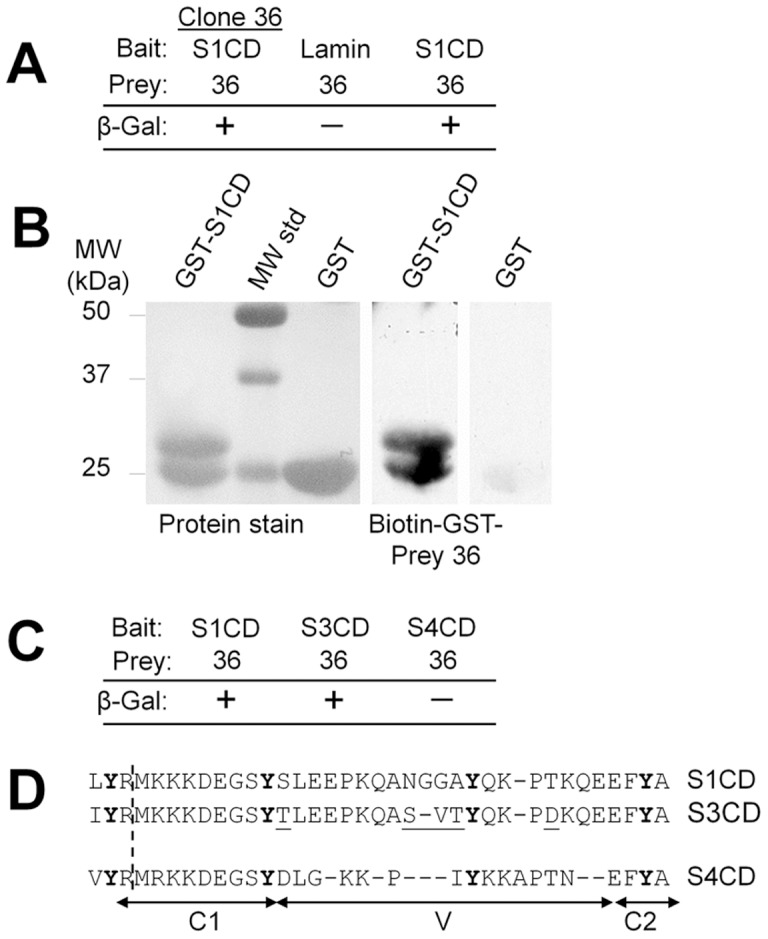
Yeast two-hybrid protein interaction assay of LexA-S1CD bait and an isolated library prey clone 36 fusion proteins. (**A**) At least two separate clones of the original library co-transformant and two-hybrid clones reconstituted by transfection of yeast clones containing only bait plasmid with prey plasmids to be tested were streaked to selective plates and assayed for interaction between the bait/prey fusion protein pairs using β-galactosidase activity as a reporter. The co-transformant clone isolated from library screening (left, Clone 36), was cured to select clones that retained only the prey vector or the bait vector. Resultant prey clones were re-transformed with an irrelevant control bait (middle, Lamin) and a clone with the S1CD bait vector was assayed after a reciprocal transformation with the clone 36 prey vector (right). (**B**) Ligand blotting of GST-S1CD with biotinylated GST-Prey 36 protein (Topors amino acids 398-516) fusion protein. Bacterially-expressed glutathione S-transferase (GST) and GST-S1CD were purified by affinity chromatography, run on SDS-PAGE gels (Coomassie blue-stained lanes on left) and blotted to nitrocellulose. The same lanes probed with biotinylated GST-Prey 36 (Topors 398–516) fusion protein (right lanes) indicate a selective interaction with GST-S1CD, which consistently ran as a double band on SDS-PAGE gels. (**C**) As in Fig. 1A, a co-transformant clone isolated from library screening was cured to select clones that retained only the prey vector, then re-transformed with bait vectors for expression of LexA-S1CD (left), LexA-S3CD (middle) and LexA-S4CD (right). (**D**) Aligned sequences of the cytoplasmic domains of Sdc-1 (S1CD), Sdc-3 (S3CD) and Sdc-4 (S4CD), with conserved domains (C1, C2) and the variable domains (V) delineated. Amino acids sequence differences between S1CD and S3CD are underlined.

A size-restricted (∼500-bp) mouse embryo VP16 fusion library (5.8×10^6^ transformants) was screened for interaction with the LexA-S1CD fusion protein in a yeast two-hybrid protein interaction assay. Co-transformed amplified clones with high β-galactosidase activity were selected and further screened to eliminate false positives. To ensure that expressed prey fusion proteins did not activate β-galactosidase in the absence of the LexA-S1CD bait fusion protein, VP16 prey clones that lacked bait plasmid were identified by loss of β-galactosidase activity after growth in selection medium containing tryptophan. These clones were then re-transformed with the pBTM116 bait plasmid to express either the original LexA-S1CD fusion protein or LexA-lamin [39], which was used as a negative control to ensure that the interaction was specific to LexA-S1CD (Fig. 1A). Clones were isolated that lost β-galactosidase activity upon loss of LexA-S1CD expression and regained β-galactosidase activity upon re-transformation to express LexA-S1CD, but that did not regain β-galactosidase activity with expression of LexA-lamin. Eight independently isolated and verified prey clones were sequenced and found to include a 356-base pair mouse cDNA encoding a 118-amino acid sequence. To confirm the interaction of S1CD with the sequence present in the isolated prey clones, biotinylated glutathione S-transferase (GST)-Prey 36 fusion protein was used to probe a blot of GST-S1CD fusion protein and, as a control, GST alone (Fig. 1B). The biotinylated probe bound GST-S1CD, which consistently ran as a doublet band, but not GST. In addition, yeast clones containing Prey 36 were co-transformed with bait plasmids for the expression of Sdc-3 cytoplasmic domain (LexA-S3CD) or Sdc-4 cytoplasmic domain (LexA-S4CD) (Fig. 1C). Interestingly, co-transformation of Prey 36 clones with LexA-S3CD bait plasmid also activated the β-galactosidase reporter, but to a significantly reduced level (a positive reaction was evident in <2 hours compared to >16 hours for LexA-S1CD and LexA-S3CD, respectively). LexA-S4CD had no activity. These data indicate that the Prey 36 sequence interacts specifically with S1CD and S3CD, with a stronger interaction evident with S1CD. Because the cytoplasmic region of Sdc-1–4 contains identical conserved domains, and the variable domains of Sdc-1 and Sdc-3 are similar, these results suggest that Prey 36 fusion protein interacts with the S1CD variable domain (see Fig. 1D for a comparison of S1CD, S3CD and S4CD amino acid sequence).

### The S1CD-binding sequence encoded by Prey clone 36 is included within murine Topors

**Figure 2:**
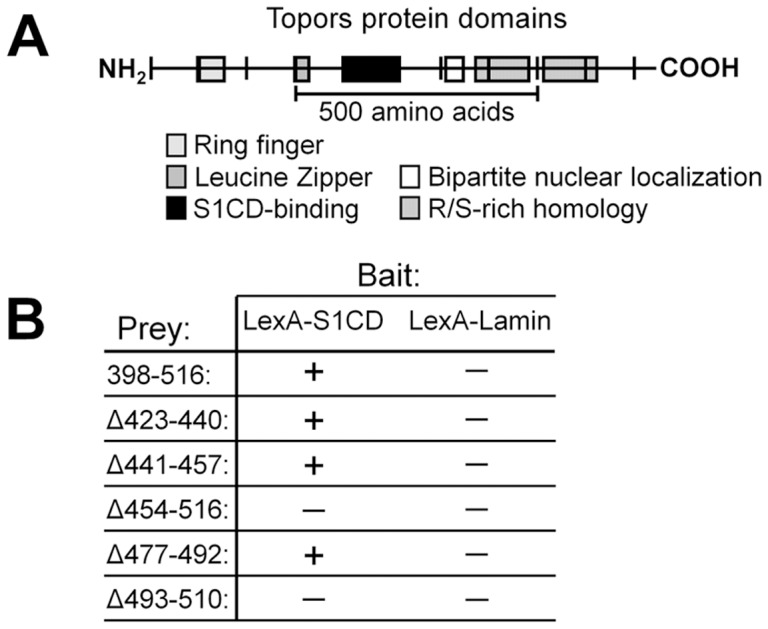
Deletion analysis of the S1CD-interacting domain of Topors. (**A**) Model of murine Topors protein domains, with the sequence corresponding to the S1CD-interacting prey 36 sequence identified by a black box. (**B**) Deletional analysis of the S1CD-binding sequence of Topors. Yeast with either LexA-S1CD or LexA-Lamin bait plasmid were transformed with either prey plasmid for the expression of complete S1CD binding sequence (Topors 398–516), or with prey plasmid expressing deletion mutants of Topors 398–516. After selection of co-transformants, interacting bait-prey pairs were identified by assay of β-galactosidase activity. Yeast two-hybrid analysis of Topors deletion mutants identifies residues 493–510 as critical for the interaction.

A search of GenBank DNA sequences indicates that the sequence isolated from the prey plasmid is included within the murine Topors coding sequence (Accession # BC040797), differing only in the substitution of A for G1351 (thus, GCT>ACT; A398>T). This substitution, which has been confirmed in all of our original clones, may represent a polymorphism. The human TOPORS sequence was published independently by two laboratories that used the yeast two-hybrid assay to screen for proteins that bind to topoisomerase I [27] or p53 [31]. The S1CD-interacting sequence of Topors that was identified in the yeast two-hybrid assay is located at residues 398-516 of the full murine 1030-amino acid sequence (see model, Fig. 2A), and is highly conserved in the human sequence (not shown). Yeast bait clones that express either LexA-S1CD or LexA-Lamin (a negative control) were co-transformed with prey vectors expressing either full length Prey 36 insert (murine Topors sequence 398–516) or various deletion mutants of that sequence (Δ423–440, Δ441–457, Δ454–516, Δ477–492, and Δ493–510) to analyze the requirements for interaction with S1CD in the yeast two-hybrid assay (Fig. 2B). Multiple clones of each co-transformant were assayed for β-galactosidase activity. As expected, transformation of LexA-S1CD with Topors 398–516 gave a positive signal, as did transformation with deletion mutants Δ423–440, Δ441–457, and Δ477–492. However, both a large (Δ454–516) and a smaller (Δ493–510) deletion in the more carboxyl terminal sequence resulted in a complete loss of β-galactosidase activity, indicating loss of interaction with the LexA-S1CD bait. No signal was produced by clones of LexA-lamin yeast co-transformed with any of the bait constructs. These results suggest that the interaction of S1CD with Topors requires amino acids 493–510 (ELVELSSDSEELGPYEKV), a sequence that contains one consensus casein 2-kinase phosphorylation site (SDSE).

### Topors interacts with Sdc-1 in biochemical and immunochemical assays

**Figure 3:**
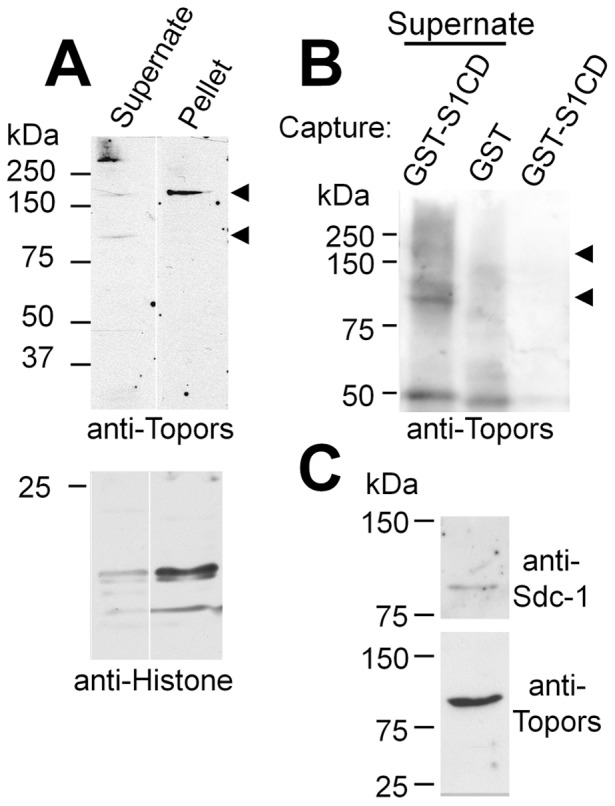
Affinity capture and western blotting of Topors. (**A**) Western blot of NMuMG cell detergent lysate (supernate) and detergent-insoluble material (pellet) probed with chicken anti-Topors antibody. Two major bands (∼110 and ∼175 kDa (arrowheads)) are apparent. The larger band is prevalent in detergent-insoluble fractions, which contain relatively more histone proteins (lower blot), consistent with enhanced presence of nuclear proteins. (**B**) Capture of endogenous Topors from cell lysates by GST-S1CD. GST-S1CD or GST alone were adsorbed to glutathione-agarose beads and incubated with detergent lysates of 3T3 cells. Anti-Topors-reactive bands captured by GST-S1CD were detected in lysates of 3T3 cells that overexpress Topors (first lane, arrows), and were similar in size to the major bands seen in western blots of whole cell lysates. Similar bands were not captured by GST alone (lane 2), nor are they present in the GST-S1CD reagent (lane 3). Non-specific bands are seen at ∼55 kDa. (**C**) Co-precipitation of Topors with polyhistidine-tagged Sdc-1 isolated by metal-ion affinity chromatography. Metal ion-affinity gel bound material was isolated from detergent extracts of cells that express polyhistidine-tagged Sdc-1. Parallel samples were run on SDS-PAGE, either directly or after heparinase and chondroitinase digestion and transferred to nitrocellulose. Parallel blots were probed with chicken anti-Topors or, for digested samples, with anti Sdc-1.

Western blotting of lysates of murine cells using a chicken antibody to Topors fusion protein identified two major bands of approximately 110 kDa and 175 kDa for murine Topors in normal murine mammary gland (NMuMG) cells (Fig. 3A), 3T3 cells (not shown) and murine SMC (see Fig. S1B), consistent with recently published work [40]. However, while both bands were apparent in the soluble fraction of lysates, only the larger band was found in the insoluble fraction. The 110 KDa band (Fig. 3A, arrow) is close to the predicted MW of mouse Topors (117 kDa), while the 175 kDa band(s) may represent SUMOylated or otherwise modified Topors [35], [41], [42]. When lanes of equally-loaded detergent-soluble and -insoluble fractions were probed with a pan-histone antibody, the detergent-insoluble fraction contained more histone, indicating that the detergent-insoluble material contained a larger proportion of nuclear material, as expected, and suggesting that the more abundant and larger (175 kDa) Topors is predominantly a nuclear form.

**Figure 4:**
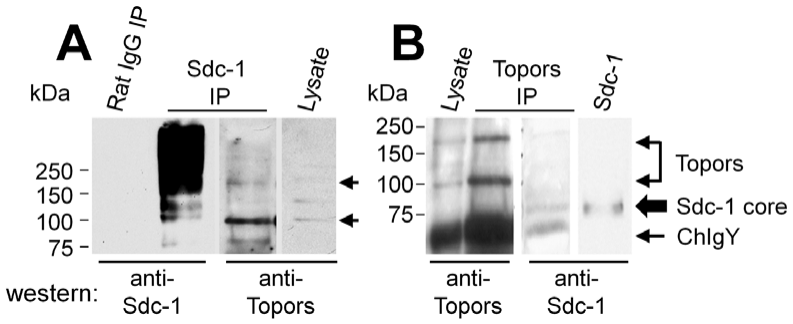
Co-immune precipitation of Sdc-1 and Topors from lysates of NMuMG cells. (**A**) Total cell lysates prepared from NMuMG cells were immune precipitated (IP) with non-immune rat IgG (lane 1) or rat anti-Sdc-1 antibody (lane 2 and 3) and then western blotted for Sdc-1 (lanes 1–2), and after stripping, re-probed with anti-Topors (lanes 3). Total cell lysate (lane 4) was included as a control for anti-Topors immunoreactivity. Arrows mark major co-precipitated Topors bands (110 and 175 kDa). (**B**) Non-immune chicken IgY-preadsorbed total cell lysates of NMuMG cells were immunoprecipitated with chicken anti-Topors, and immune precipitates were heparinase- and chondroitin ABC lyase-digested prior to SDS-PAGE and western blotting. Lysate (lane 1) and Topors immunoprecipitates (lane 2 and 3) that were probed on western blots for Topors (lanes 1 and 2) had Topors bands (110 and 175 kDa bands, double arrow), or for Sdc-1 (lane 3), which gave a band of ∼85 kDa (large arrow, lane 3 and 4), similar in size to a band present in a Sdc-1-positive control (lane 4), prepared by ion-exchange chromatography from the medium of NMuMG cultures. Lower MW heavy bands represent chicken IgY (NChIgY) from the immune precipitate (arrow). Lanes 1–3 are from the same blot with lane 3 representing a stripped and re-probed lane of the Topors immune precipitate run in parallel to that shown in lane 2. Lane 4 represents an adjacent lane from the same blot, at a longer exposure time.

Lysates of 3T3 cells that overexpress Topors were prepared and incubated with GST-S1CD or GST alone to determine if Topors could be captured by the S1CD. Western blots of GST-S1CD and GST-bound ligands probed with anti-Topors showed bands of approximately 110 and 175 kDa in the GST-S1CD lane (Fig. 3B), while non-specific bands of about 50 kDa were also present in samples incubated with GST alone or with the GST-S1CD fusion protein run directly on the gel. These data indicate that the S1CD interacts with Topors in detergent lysates of cells. Using another approach, material bound to a metal affinity resin from lysates of NMuMG cells that express polyhistidine-tagged murine Sdc-1 was digested with heparinase and chondroitinase ABC, run on SDS-PAGE, and transferred to western blots that were then probed with either anti-Sdc-1 or anti-Topors (Fig. 3C). Immunoreactive bands of Sdc-1 and Topors were recognized by the appropriate antibodies in parallel blots. Finally, total cell lysates from NMuMG cells were prepared and either Sdc-1 (Fig. 4A) or Topors (Fig. 4B) was immunoprecipitated. Both low and high MW Topors present in cell lysates (Fig. 4A, lane 4) co-precipitated with Sdc-1 (Fig. 4A, lane 3, arrows), while undigested Sdc-1 ran as a broad, high MW band (Fig. 4A, lane 2). Conversely, Sdc-1 co-precipitated with Topors and ran as a core protein of 80–85 kDa after digestion to remove Sdc-1 glycosaminoglycan chains (large arrow, Fig. 4B). These bands were not seen in immune precipitations carried out with preimmune chicken IgY or normal rat IgG. The heavily stained lower MW bands in Topors immunoprecipitates correspond to chicken IgY released with immunoprecipitated material. Taken together, these results confirm an interaction between Topors and S1CD within mammalian cells, as predicted by the yeast two-hybrid assay.

### Topors localizes to the nucleus and cell periphery in murine epithelial cells and tissue

**Figure 5:**
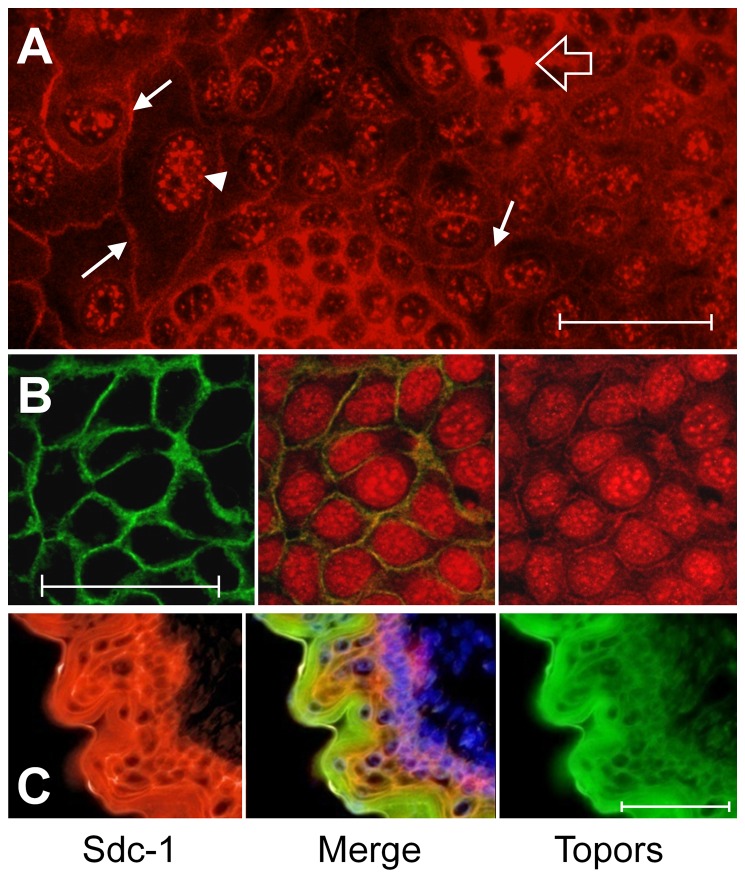
Immunolocalization of Topors and Sdc-1. (**A**) Immunofluorescent staining of confluent NMuMG cells demonstrates that Topors is present in a punctate pattern in nuclei (e.g., arrowhead), consistent with previous reports of nuclear localization. In addition, Topors immunoreactivity is present at cell boundaries (arrows) as well as heavily present in the cytoplasm of cells undergoing mitosis (open arrow). Bar  = 20 μm. (**B**) Immunofluorescent images of a single field of confluent NMuMG cells immunostained for mouse Sdc-1 (green, left) and Topors (red, right), with a merged image presented in the middle panel. Bar  = 20 μm. (**C**) Immunofluorescent staining of dorsal epidermis of an e13.5 mouse embryo for Sdc-1 (red, left) and Topors (green, right). The merged imaged (center) includes a nuclear stain (DAPI, blue). Bar  = 50 μm.

Topors and Sdc-1 in NMuMG cells and in mouse embryonic epithelium were localized by immunofluorescent staining to determine if they co-localize at specific cellular sites. In NMuMG cells (Fig. 5A), Topors clearly localized to punctate sites in nuclei, in agreement with previous studies [27], [43] showing that Topors is present in nuclear pro-myelocytic nuclear (PML)-bodies [43]. However, Topors was also present at the cell periphery (Fig. 5A, arrows) and in the cytoplasm of mitotic cells (Fig. 5A, large arrow). This is a distribution similar to that recently reported in ciliated cells, particularly in the retinal epithelium [40]. Staining for Topors at juxtamembranal sites is similar to the basolateral distribution of Sdc-1 in these cells, with which Topors immunostaining overlaps (Fig. 5B). Immunofluorescent staining for both Sdc-1 and Topors in sections of embryonic murine dorsal epidermis (Fig. 5C) also indicate that Topors is present in both the nucleus and cytoplasm, and that it co-localizes with Sdc-1 at sites near the cell surface. Nuclear staining for Topors was most apparent in basal cells of the epithelium, and was absent in stratified epithelial cells superficial to the basal layer and near the very attenuated, strongly autofluorescent corneal layer. The distribution of both Sdc-1 and Topors near the cell periphery suggests that an interaction between these molecules may occur at the juxtamembranal region of the cytoplasm.

### Topors is a growth promoter for arterial smooth muscle cells and is required for the inhibitory effect of Sdc-1 on SMC proliferation and PDGF-B induction

**Figure 6:**
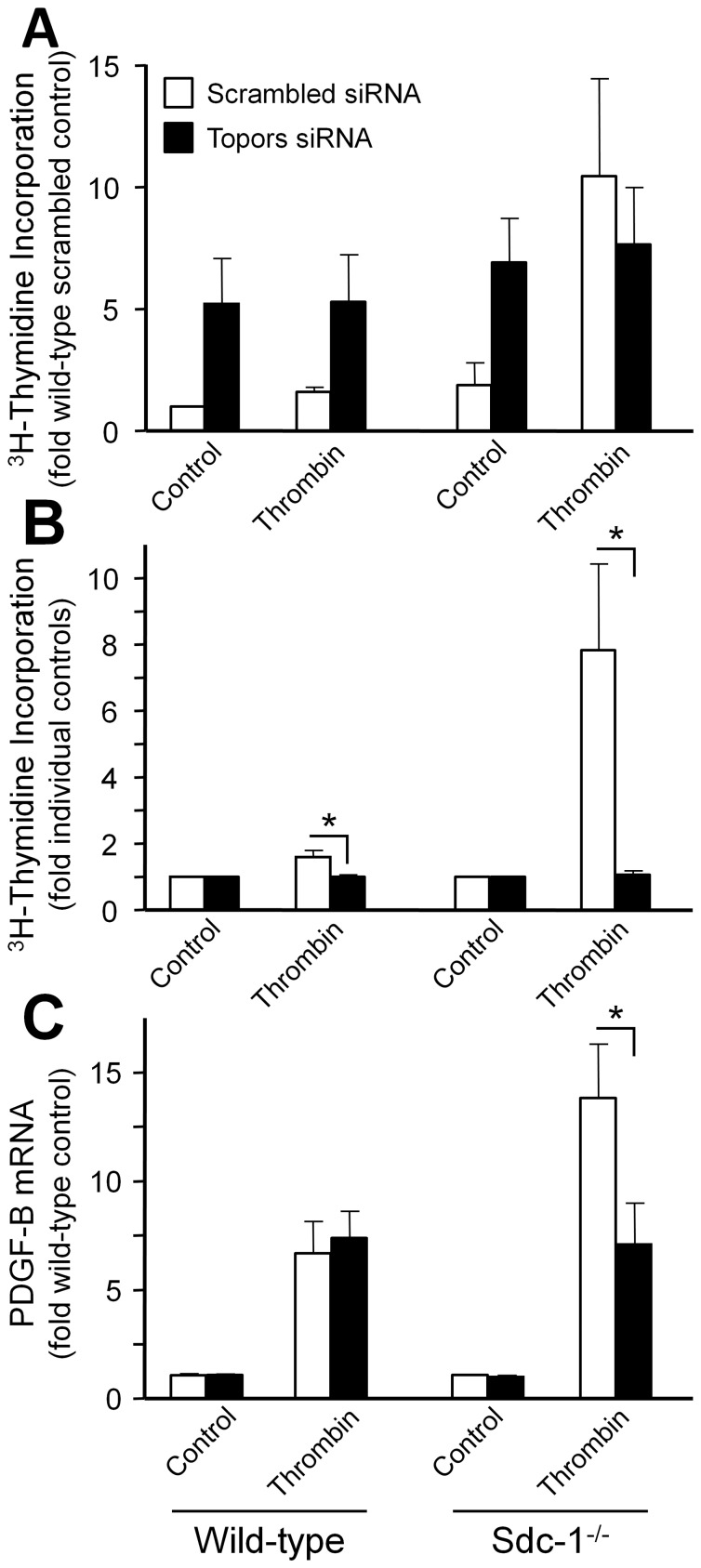
The effect of siRNA-mediated Topors knockdown on cell proliferation and PDGF-B chain expression by wild-type and Sdc-1 null SMCs. (**A**) The effect of siRNA to Topors (closed bars) or a scrambled control (open bars) on [^3^H]-thymidine incorporation mediated by thrombin in wild-type and Sdc-1 null SMCs. Values are the mean ± SEM of four experiments expressed as the fold of wild-type scrambled siRNA control. (**B**) The same data as in section (A) except that data is expressed as the fold of its appropriate control (either the scrambled control or the siRNA to Topors of either wild-type or Sdc-1 null). (**C**) The effect of siRNA to Topors (closed bars) or a scrambled control (open bars) on PDGF-B chain mRNA mediated by thrombin in wild-type and Sdc-1 null SMCs. Values are the mean ± SEM of four experiments for thrombin expressed as the fold of wild-type scrambled siRNA control. * P<0.05.

We have shown that arterial SMCs from Sdc-1 null mice proliferate more in response to various growth factors, including thrombin, when compared to wild-type SMCs [36]. Whether interaction of Sdc-1 with Topors is involved in this effect of Sdc-1 in SMCs is not known. Therefore, we determined the effect of knocking down Topors on thrombin-induced entry into S phase in wild-type and Sdc-1 null SMCs. As shown previously, treatment with thrombin increased ^3^H-thymidine incorporation into DNA more in Sdc-1 null SMCs than in wild-type SMCs (7.8 vs. 1.6 fold, respectively; open bars, Fig. 6A). Knockdown of Topors in control and thrombin-treated SMCs was effective as demonstrated by a decrease of 70–80% in Topors mRNA (Fig. S1A), and in both prominent Topors protein bands (Fig. S1B). Loss of Topors increased basal DNA synthesis, but blunted the stimulatory effect of thrombin and abolished the inhibitory effect of Sdc-1 on thrombin-mediated entry into S phase (Fig. 6A). This is more easily seen by normalizing values to the appropriate control so that the thrombin-dependent response is clear (Fig. 6B). We previously observed that wild-type SMC proliferation was not dependent on endogenous PDGF-B, but that the increased proliferation of Sdc-1 null SMCs in response to FBS, PDGF-BB, and thrombin was dependent on endogenous PDGF-B [36]. We have confirmed that Sdc-1 null SMCs induce PDGF-B in response to thrombin to a greater degree than wild-type SMCs (Fig. 6C). In addition, while loss of Topors had no effect on PDGF-B induction by thrombin in wild-type SMCs, loss of Topors abolished the effect of Sdc-1 deficiency on thrombin-mediated PDGF-B induction (Fig. 6C). These data indicate that the decreased proliferation observed after knockdown of Topors in wild-type SMCs (Fig. 6B) is independent of PDGF-B, but that part of the inhibition of thrombin-mediated proliferation caused by knockdown of Topors in Sdc-1 null SMCs is dependent on endogenous PDGF-B production.

## Discussion

We have identified Topors, an unusual dual function ubiquitin and SUMO-1 E3 ligase, as a novel ligand of the Sdc-1 cytoplasmic domain. In addition, we have found that Topors is a promoter of SMC proliferation which, in the absence of Sdc-1, stimulates growth partly through increased production of the growth factor, PDGF-B. Conversely, in the absence of the growth and PDGF-B-inducing activities of Topors, the inhibitory effect of Sdc-1 on growth and PDGF-B [36] is not observed. The simplest hypothesis to explain these data is that the S1CD binds Topors and prevents its growth-promoting activities either by directly binding and inhibiting the ligase activity of Topors, or by preventing it from acting at another location. In this regard, we found by deletional mutagenesis that the sequence E493LVESSDSEELGPKYVE510 of Topors is required for the interaction with Sdc-1. This sequence is N-terminal to the region of Topors that alters tumor cell growth (aa 540–704; [24], [44]) and interaction with p53 [35] as well as to S718, phosphorylation of which regulates Topors-dependent modification of p53 [34]. However, it overlaps with the sequence that mediates localization of Topors to nuclear speckles (aa 437–574) and Topors SUMOylation activity [42], suggesting that Sdc may modify these activities.

In apparent contrast to our results, several studies have reported that downregulation of Topors or Sdc-1 is correlated with increases in cellular growth or transformation. For example, Topors expression is reduced in malignant tissues and transformed cell lines, and overexpression of Topors suppresses tumor cell growth by increasing p53 activity [24], [44],[45]. Antisense-mediated Sdc-1 downregulation promotes the transformation of mammary epithelial cells, and induces attachment-independent cell growth and tumorigenesis in nude mice [37]. Moreover, Sdc-1 expression is suppressed during the steroid-induced conversion of S115 mouse mammary tumor cells from an epithelioid to a fibroblastoid morphology [46]. However, juvenile Sdc-1 null mice have an increased resistance to tumor development. Such differences in the effect of Topors may be explained by differences between regulatory factors in normal and malignant cells for Topors, which can both stabilize p53, perhaps by SUMOylation [42], and destabilize p53 via Topors-dependent ubiquitinization [47].

In addition to its effects on cell proliferation, Sdc-1 influences cell migration and cell death. These varied effects may result from the diversity of factors and signaling cascades that are influenced by Sdc-1, including extracellular matrix proteins and various receptors such as FGFR, Wnt and hedgehog signaling [48], [49], as well as integrins [50]. Chen and his colleagues reported that Sdc-1 keeps β1 integrin in the activated conformation and inhibits cell migration [51]. Activation of β1 integrin also inhibits SMC growth [52]. Increased Sdc-1 expression levels correlate with resistance to UV irradiation-induced apoptosis in a virally-transformed human fibroblast cell line [38]. In addition, long-chain, polyunsaturated fatty acid-mediated cell death requires Sdc-1 and the Sdc-1 ectodomain can cause apoptosis of prostate cancer cells [53]. The intracellular signaling pathways mediating these effects have not been fully investigated. The ubiquitin and SUMO E3 ligase, Topors, binds and modifies several important cellular regulatory proteins, including p53 [31], NKX3.1 [26], mSin3A [54], DJ-1 [29], and topoisomerase I [27], the activity of which is modified in *in vitro* assays by heparan sulfate and FGF-2 [55]. In addition, there are a number of other potential targets for Topors that are related to chromatin modification or transcriptional regulation [54]. A possible connection between Topors and Sdc-1 regarding receptor signaling was suggested by Chakarova and colleagues [56], who proposed that retinal developmental defects associated with Topors deficiency may result from defective Wnt and hedgehog signaling, both of which are regulated by Sdc-1 [48], [49]. Thus, Sdc-1 and Topors may regulate cellular growth via linked signaling pathways.

The observation that Sdc-1 binds Topors provides a link between Topors and Sdc-1, but the inhibitory effects of Sdc-1 and the stimulatory effects of Topors on wild-type SMC DNA synthesis may be independent. Because we have previously demonstrated that the augmented thrombin-mediated DNA synthesis and proliferation observed in Sdc-1 null SMCs is mediated by endogenous PDGF-B [36], and we now show that the augmented thrombin-mediated PDGF-B induction in Sdc-1 null SMCs is dependent on Topors, we conclude that PDGF-B-dependent portion of thrombin-mediated proliferation of Sdc-1 null SMCs is mediated by Topors. While we have confirmed the finding from our previous report [36] that PDGF-B induction by thrombin is increased in Sdc-1 null SMCs, other growth factors such as FGF2 and EGF have a greater proliferative effect in Sdc-1 null SMCs in a manner independent of PDGF-B [36]. It is not known if Topors is required for SMC proliferation mediated by these other factors, nor is it known if TOPORS plays a role in the effect of Sdc-1 on vascular injury *in vivo*. Of interest, TOPORS expression is the same in carotid arteries of wild-type and Sdc-1 null mice and is not altered 4 or 7 days after carotid ligation injury in either wild-type or Sdc-1 null mice (unpublished microarray experiments). Thus, the extent to which the Sdc-1 phenotype depends on Topors is not yet clear.

Our studies demonstrated a high MW (∼175 kDa) form of Topors that was previously reported for human Topors [42], [43] and that may represent a form modified by SUMOylation. The additional major band of ∼110 kDa (Figs. 3, 4 and S1B) that is close in size to the ∼117 kDa predicted MW of full-length murine Topors was recently noted in western blots of murine cells using a different anti-Topors antibody [40] and may represent a relatively unmodified form. Alternatively, the lower MW band may represent a splice variant corresponding to human TOPORS isoform 2 in which in amino acids 169–366 are deleted. Of particular interest, the lower MW Topors is the principal form that co-precipitates with Sdc-1 (Fig. 4A) and was found primarily in the detergent-soluble, histone-poor, fraction of cell extracts, which is consistent with the primary site of Topors interaction with Sdc-1 being the non-nuclear fraction. This is also consistent with the co-localization of these molecules at the periphery of NMuMG cells. Topors was also present in a speckled pattern in the nuclei of epithelial cells, in agreement with other studies that localized fluorescent protein-tagged and endogenous Topors in nuclear PML-bodies [27], [42], [43], consistent with the presence of a bipartite nuclear localization domain in Topors. While we did not detect Sdc-1 immunoreactivity at nuclear sites, heparan sulfate and heparan sulfate proteoglycans, including Sdc-1 [57], [58], [59], have been localized to the nuclei of cells, as well as to spindle structures and tubulin [60]. Topors has also been shown to be associated with these same locations [40]. Internalization and trafficking of Sdc-1 to the nucleus can regulate the accumulation of its ligand, FGF-2, in the nucleus [61], [62]. Moreover, other intracellular ligands of Sdcs, such as syntenin/mda-9 [63] and CASK/LIN [18] have been shown to translocate between nuclear and cytoplasmic compartments, a process that may occur concurrently with the internalization of Sdc-1 via endocytotic vesicular uptake. In this regard, Sdc-1 has been shown to bind to Rab5, a regulator of early endosome fusion [36], [64]. Also, CASK/LIN, which binds to the C2 domain of the S1CD, has been reported to both increase and decrease cell proliferation [7] in different systems [65]. However, the finding that the S3CD interacts weakly with Topors and the S4CD does not, suggests that the variable domain of Sdc-1 interacts with Topors, since the S3CD differs from the S1CD by only 5 amino acids (Fig. 1). Interactions between S1CD and Topors may not, therefore, be limited to the juxtamembranal cytoplasmic compartment, but may also occur in endocytotic vesicles.

The observation from this work that the S1CD binds Topors indicates a potential pathway by which previously observed Sdc-1-dependent effects on cellular growth, in part via PDGF-B, are mediated. However, the specific mechanisms involved in the control of these cellular processes remain to be clarified.

## Materials and Methods

### Cells and cell culture

The 3T3 D1 cell line, provided by Dr. Daniel Bowen-Pope (University of Washington, Seattle, WA), was derived by subcloning NIH 3T3 cells, purchased from ATCC (www.atcc.org). Normal murine mammary gland (NMuMG) cells were purchased from American Type Culture Collection (ATCC). Arterial smooth muscle cells (SMCs) were isolated from adult male C57BL/6 mouse aortae, as described [36]. Cells were characterized as SMCs by smooth muscle α-actin expression, and used up to the thirteenth passage. SMCs isolated from litter-mate Sdc-1 null mice did not express Sdc-1 mRNA or protein [36]. All cells were cultured in Dulbecco's modified Eagle's Medium (DMEM, GIBCO Cell Products, Invitrogen Technologies) with 10% fetal bovine serum (Atlanta Biologicals), non-essential amino acids, pyruvate and glutamate (GluMax), all from GIBCO/Invitrogen.

### Yeast two-hybrid protein interaction assay

The yeast two-hybrid transcriptional reporter selection system was used as originally described and modified [66], [67] to screen a size-selected (∼500-bp) mouse embryo (9.5–10.5 d.p.c.) cDNA-fusion library in the pVP16 plasmid (kindly provided by Dr. Stanley Hollenberg, University of Washington) for interaction with the 36 carboxyl-terminal amino acids of mouse Sdc-1 (S1CD). A 252-base pair cDNA encoding S1CD (a gift of Dr. Markku Jalkanen, Turku Centre for Biotechnology, University of Turku, Finland) was fused in-frame to the cDNA encoding the first 211 amino acids of the LexA protein in the expression vector pBTM116. VP16 library clones that interacted with LexA-S1CD fusion protein were identified by the activation of transcriptional reporters for both histidine prototrophy and β-galactosidase activity [68] in *S. cerevisiae* strain L40 clones isolated by growth under 5-fold selective conditions (growth in defined medium lacking tryptophan, leucine, lysine, uridine and histidine). Other LexA-fusion protein constructs were used to screen selected VP16 clones for specificity of interaction with the LexA-S1CD fusion protein. These included LexA-lamin C fusion protein, plasmid pLam5 [67], LexA-Sdc-3 cytoplasmic domain (S3CD) and LexA-Sdc-4 cytoplasmic domain (S4CD). LexA-S3CD was constructed from a pCMV5neo vector containing the complete coding sequence of Sdc-3 [69] (provided by Dr. David J. Carey, Geisinger Clinic, Danville, PA) by excising a 851 bp PstI restriction fragment containing the S3CD and sequence 3′ to the stop codon and ligating in frame into the multiple cloning site of pBMT116. Similarly, S4CD (bp 527 to bp 621) was amplified with a high fidelity polymerase (Phusion, Finnzymes, New England Biolabs) from full-length Sdc-4 in pcDNA3 vector (Invitrogen Technologies) [70] that was provided by Dr. John Couchman (Department of Biomedical Sciences, University of Copenhagen, Copenhagen, Denmark) using forward primer 5′cgggatcc-tggtgtaccgcatgaagaagaag3′ and reverse primer 5′cgggatcc-tcatgcgtagaactcattggtg3′ with attached BAMHI site sequences. Purified S4CD cDNA was ligated into the BAMHI restriction site of pBTM116 vector. All plasmid inserts were sequenced prior to use. To test for specificity of interaction and to eliminate false positives, two additional tests were performed. First, putative S1CD interactive VP16-library clones were cured of the BTM116 bait vector by selection for loss of tryptophan prototrophy after growth in non-selective media. Cured clones were then assayed for β-galactosidase activity to ensure that the fusion protein expressed by the library clone alone could not activate the transcriptional reporter. Second, library clones that could not independently activate the transcriptional reporter were then re-transformed with either pBTM116-LexAS1CD, or an irrelevant control bait plasmid (pBTM116-LexALam5) that expresses a lamin C fusion protein [39], [66]. Co-transformant clones were then tested for β-galactosidase activity to determine if activation of transcriptional activity for the reporter was specific for interaction with LexA-S1CD. cDNA from pVP16 library clones was amplified using the polymerase chain reaction (PCR) [71]. The 5′ oligonucleotide primer is derived from sequence upstream of the cloning site within the VP16 vector (5′-GAG TTT GAG CAG ATG TTT A-3′) and the 3′ oligonucleotide primer is the forward M13 universal primer (5′-GTT GTA AAA CA CGG CCA GT-3′) that is cloned in the reverse orientation at the 3′ end of the VP16 sequence within the VP16 vector. DNA sequencing was performed by the DNA Sequencing Facility, Dept. of Biochemistry, University of Washington, Seattle, WA.

### Deletion mutagenesis of S1CD-binding sequence of murine Topors

Deletion mutants of Topors 398–516 in VP16 vectors were constructed using a site-directed mutagenesis kit (QuikChange II, Stratagene). Primers were designed to delete amino acids 423–440, 441–457, 454–516, 477–492, or 493–510 from the sequence of the isolated library clone (Topors 398–516), using the Stratagene (http://labtools.stratagenecom/QC) website. Topors 398–516 VP16 plasmid was used as a template, and mutant primers were used to amplify deletion mutants using high fidelity PfuUltra HF polymerase (Stratagene). After removal of template plasmid by restriction enzyme digestion, according to the manufacturer's protocol, the resulting mutant plasmids were isolated from transformed bacterial colonies and sequenced.

### Preparation of antibodies against the GST-Topors fusion protein

The 356-bp library cDNA (Topors 398–516) was ligated into the NotI site of pGEX4T1 vector (Amersham Pharmacia Biotech), and the sequence of the insert was confirmed by sequencing. GST-fusion protein synthesis was induced in vector-transformed DH5α *E. coli* (Stratagene) with 1 mmol/L isopropyl-β-D-thiogalactoside and isolated from clarified sonicates of the cells by adsorption to glutathione-agarose (Sigma-Aldrich), according to the manufacturer's instructions. For the production of chicken polyclonal antibodies, purified GST-Topors 398–516 (800 µg) was supplied to Flock Antibodies, Spokane, WA to inoculate hens from which pre-immune eggs had been collected. Chicken IgY was prepared from eggs collected by Flock Antibodies by precipitation with polyethylene glycol from chloroform-extracted yolks [72], dissolved in phosphate buffered saline, and stored at –20°C in 50% glycerol. Antibody specificity was confirmed by western blotting purified antigen and whole murine cell lysates.

### Ligand blotting binding assay and capture of endogenous proteins by GST-fusion proteins

To prepare GST-S1CD, mouse S1CD sequence excised from the EcoRI-SalI restriction site of the pBluescript vector was directionally cloned into the multicloning site of the pGEX4T1 vector as described above for GST-Topors 398–516. After transfection of the vector into BL21 *E. coli* (Stratagene), plasmid DNA from selected clones was isolated and the inserts were confirmed by sequencing. GST and GST-S1CD fusion proteins were expressed and purified by affinity chromatography as described above, and 1–10 µg/lane were run on 10% reducing SDS-PAGE gels and transferred to nitrocellulose. Blots were blocked with 2% bovine serum albumin (BSA) in Tris-buffered saline (TBS) with 0.1% Tween-20 and probed with GST-Topors 398–516 that was biotinylated using NHS-Biotin (Thermo Fisher Scientific), following the manufacturer's instructions. Washed blots were incubated with horseradish peroxidase conjugated-NeutrAvidin (Invitrogen), and bound biotinylated ligand was visualized by enzyme-linked chemiluminescence using SuperSignal West Dura substrate (Thermo Fisher Scientific). To capture Topors from murine cell extracts, GST and GST-S1CD, prepared as described above, were dialyzed against PBS, and diluted to 50–100 µg/mL in 50% glycerol. Ten µg of each purified GST protein was adsorbed onto 200 µL of washed glutathione-agarose overnight at 4°C, washed thrice with PBS, and then with NET-BSA (50 mmol/L Tris-HCl, pH 7.5 with 1% NP-40, 1 mmol/L EDTA and 0.25% BSA), before resuspension in NET-BSA. To prepare 3T3 cells that overexpress murine Topors, full-length murine Topors was excised from IMAGE clone 3492178 (bp 1-3211 of Genbank BC040797) with SmaI and DrdI, and blunt-ended with T4 DNA polymerase, which resulted in a 14 amino acid truncation of the carboxyl-terminus. This sequence was then cloned into the SalI site of the pCMV-Tag1 vector (Stratagene), which had been previously modified by deletion of the FLAG epitope tag, to yield a vector with a carboxyl-terminal c-Myc epitope-tagged Topors. Cells were transfected using Fugene 6 (Roche Molecular Systems) and selected in medium with 0.6 mg/mL G418 (Sigma-Aldrich). The presence of RNA encoding the transgene was confirmed by PCR amplification from reverse-transcribed total RNA, and expression of protein was determined by western blots of cell extracts. Confluent 3T3 cells (1×107) were washed twice with PBS at 4°C, scraped into ice-cold PBS and pelleted by centrifugation. Cell pellets were extracted with triple detergent buffer [41] (50 mmol/L Tris-HCl, pH 8.0, with 150 mmol/L NaCl, 1% NP-40, 0.5% deoxycholate, 0.1% SDS and 2 mol/L urea) with protease inhibitors at 4°C and diluted with NET-BSA with protease inhibitors. Extracts were clarified by centrifugation and incubated with constant mixing at 4°C with 50 µL of glutathione-agarose with preadsorbed GST protein for 2 hours. After incubation, glutathione-agarose was washed twice with NET-BSA and thrice with PBS, before elution of GST-proteins and endogenous captured proteins with 10 mmol/L reduced glutathione in 50 mmol/L Tris-HCl, pH 8.

### Co-immunoprecipitation, affinity-coprecipitation, and western blotting

For immunoprecipitation of endogenous Sdc-1 and Topors from NMuMG cells, cell lysates were sonicated, clarified by centrifugation, and diluted in NET-BSA. In some experiments, cells were incubated for 10 minutes at 4°C with 1% NP-40 in PBS containing a protease inhibitor cocktail before scraping cells from plates and obtaining detergent-soluble supernatants and insoluble pellets after centrifugation at 10,000xg. Insoluble material was solubilized by sonication and boiling in 5% SDS. For immune precipitations, cell lysates were incubated with either non-immune chicken IgY or rat IgG (Sigma-Aldrich) overnight at 4°C before precipitation of non-specific complexes with either rabbit anti-chicken or rabbit anti-rat antibodies (Jackson ImmunoResearch) bound to rProteinA-Agarose (RepliGen). Non-specific immunoprecipitates were eluted from the rProteinA-Agarose in a low pH elution buffer (Thermo Fisher Scientific), neutralized, and dialyzed. Lysates that had been previously cleared with non-specific IgG were then incubated with specific primary antibodies (chicken anti-Topors or rat anti-mouse Sdc-1 (281-2, BD Pharmingen)) and immune precipitates were prepared as described above. Prior to electrophoresis, some immune precipitates were digested with a mixture of 5 U/mL each of heparin lyases I, II and III (Sigma-Aldrich) and 5 U/ml chondroitin ABC lyase (Sigma-Aldrich) in Tris-HCl, pH 7.3 with 15 mmol/L sodium acetate and 10 mmol/L calcium chloride for 3 hours at 37°C. Samples were boiled in reducing sample buffer and run on 8% SDS-PAGE minigels. Prestained MW standards (Precision Plus Dual Color, BioRad) were used to estimate band MW. For western blotting, SDS-PAGE gels were equilibrated in transfer buffer [73], and transferred to nitrocellulose (BA83, Schleicher and Schuell Bioscience). Transfer membranes were blocked with 2% bovine serum albumin (Fraction V, Boehringer Mannheim Corp.) in Tris-buffered saline with 0.1% Tween-20, and probed with either 0.1 μg/mL of rat anti-mouse Sdc-1 antibody (281–2) or 1 μg/mL chicken anti-Topors antibody in blocking buffer for 4 hours at room temperature, followed by either horseradish peroxidase-conjugated goat anti-rat light chain-specific IgG, or donkey anti-chicken IgY (H+L) (AffiniPure, Jackson ImmunoResearch), respectively. Blots were developed using the peroxidase enzyme-linked chememiluminscence substrate, SuperSignal West Dura (Thermo Fisher Scientific). Metal ion-affinity chromatography was used to isolate polyhistidine-tagged Sdc-1 from cell lysates of 3T3 cells transfected with a vector containing polyhistidine-tagged full-length mouse Sdc-1 sequence. To construct the expression vector, full-length mouse Sdc-1 (bp 223–1196 of Genbank NM011519) was excised from a pTRE-Syn1 construct (a kind gift of Dr. David Graves and Dr. Helene Sage, Benaroya Research Institute at Virginia Mason, Seattle) with NotI and XbaI and inserted directionally into corresponding NotI and XbaI sites in pcDNA6HISA (Clontech). Cells were transfected using Fugene 6 (Roche Molecular Systems) and selected in medium with 1 μg/mL blasticidin (Clontech). Cell lysates were prepared by extraction with triple detergent buffer containing protease and phosphatase inhibitors, essentially as described above. Extracts were incubated overnight with TALON metal affinity resin (Clontech) at 4°C, which was then washed in extract buffer. Bound proteins were eluted in extract buffer with 150 mmol/L imidiazole. Electrophoresis of eluted proteins on SDS-PAGE, transfer and western blotting were carried out as described above.

### Immunocytochemical staining

Sagittal sections of methyl Carnoy's fixed and paraffin-embedded mouse embryos were a gift E. H. Sage, Benaroya Research Institute, Seattle WA, and consisted of unused tissue collected under a protocol approved by the Benaroya Research Institute Animal Care and Use Committee. Sections were prepared, de-paraffinized, rehydrated, and subjected to epitope retrieval using 10 mmol/L citrate buffer, pH 6 at 80°C for 10 minutes, followed by blocking in 2% BSA in PBS, pH 7.2. Indirect immunofluorescent staining of mouse embryonic sections and cultured NMuMG cells, fixed and blocked as above, for Sdc-1 or Topors was accomplished using a rat monoclonal antibody against murine Sdc-1 (0.5 μg/mL, 281–2) or 2–4 μg/mL chicken anti-Topors, respectively, followed by the appropriate Alexafluor 488- or Alexafluor 546-tagged secondary antibodies (Molecular Probes, Invitrogen) at 1–2 μg/mL in blocking buffer with or without 1 μg/mL 4′,6-diamidino-2-phenylindole (DAPI, Sigma-Aldrich) to label cell nuclei. After extensive washing with PBS, samples were mounted using Gel/Mount (Biomedia Corp.). Non-immune chicken IgY or rat IgG was used as a negative control for the primary antibody on parallel sections. Images of co-immunofluorescent stained tissue and cells were acquired and merged on a Leica DMIRB inverted microscope, which was equipped for fluorescent epi-illumination, using a Diagnostic Instruments Pursuit 4.0 megapixel chilled color CCD camera and Spot software, version 4.5.9.1.

### Transfection of siRNA to target murine Topors in murine smooth muscle cells

The siRNA target sequence for Topors knockdown was GCT AGA TCT CCC TTC AAT ATG (bp1234–1254 of GenBank #BC040797). Synthesis and purification was with a Silencer siRNA Construction Kit (Ambion). For DNA synthesis experiments, SMCs were suspended in 10% FBS and seeded at 6250 cells per cm^2^, before transfection of the cells with Topors or scrambled siRNA (30 μmol/L) using HiPerFect Transfection Reagent (Qiagen) according to the manufacturer's instructions. The next day, medium was changed to serum-free DMEM, left overnight, and changed again. After 2 days, 10 nM human α-thrombin (American Diagnostica) was added. For DNA synthesis experiments, 0.5 μCi/mL [^3^H]-thymidine was also added and after 24 hours DNA synthesis was determined in triplicate samples as previously described [74]. Cells were harvested after 4 hours for determination of PDGF-B mRNA by quantitative reverse-transcriptase polymerase chain reaction (qRT-PCR) as previously described [36]. For these experiments, significant differences were determined by use of a two-tailed Mann-Whitney test. In addition, some samples were extracted, subjected to 8% SDS-PAGE, and transferred to nitrocellulose membranes, upon which immunodetection of Topors was performed using a chicken anti-GST-murine Topors (398–516) antibody, prepared as described above, horse-radish peroxidase-conjugated donkey anti-chicken secondary antibody (Jackson Immunochemicals) and enhanced chemiluminescence (Thermo Fisher Scientific). Separate samples were used to extract RNA for qPCR for Topors that was done using the Applied Biosystems Taqman probe for Topors (Assay# Mm00506480_m1).

## Supporting Information

Figure S1 **The effect of siRNA-mediated Topors knockdown on Topors mRNA and protein levels in SMCs.** (**B**) Topors mRNA expression was determined by qPCR in samples from untreated control cultures and in cultures 48 hours after transfection with scrambled or Topors-targeted siRNA, as determined in a representative experiment with triplicate cultures. (**C**) Western blotting for Topors in total cell lysates from cultures of untreated Sdc-1 null cultures and cultures 48 hours after transfection with scrambled or Topors-targeted siRNA. Under these conditions Topors siRNA treatment resulted in a ∼70% Topors mRNA and protein knockdown.
(TIF)
