# Supplementary figures and images for: Inhibition of PDGF-B Induction and Cell Growth by Syndecan-1 Involves the Ubiquitin and SUMO-1 Ligase, Topors
Source: PLoS One. 2012 Aug 17;7(8):e43701. doi: 10.1371/journal.pone.0043701 (PMC3422340; doi:10.1371/journal.pone.0043701)

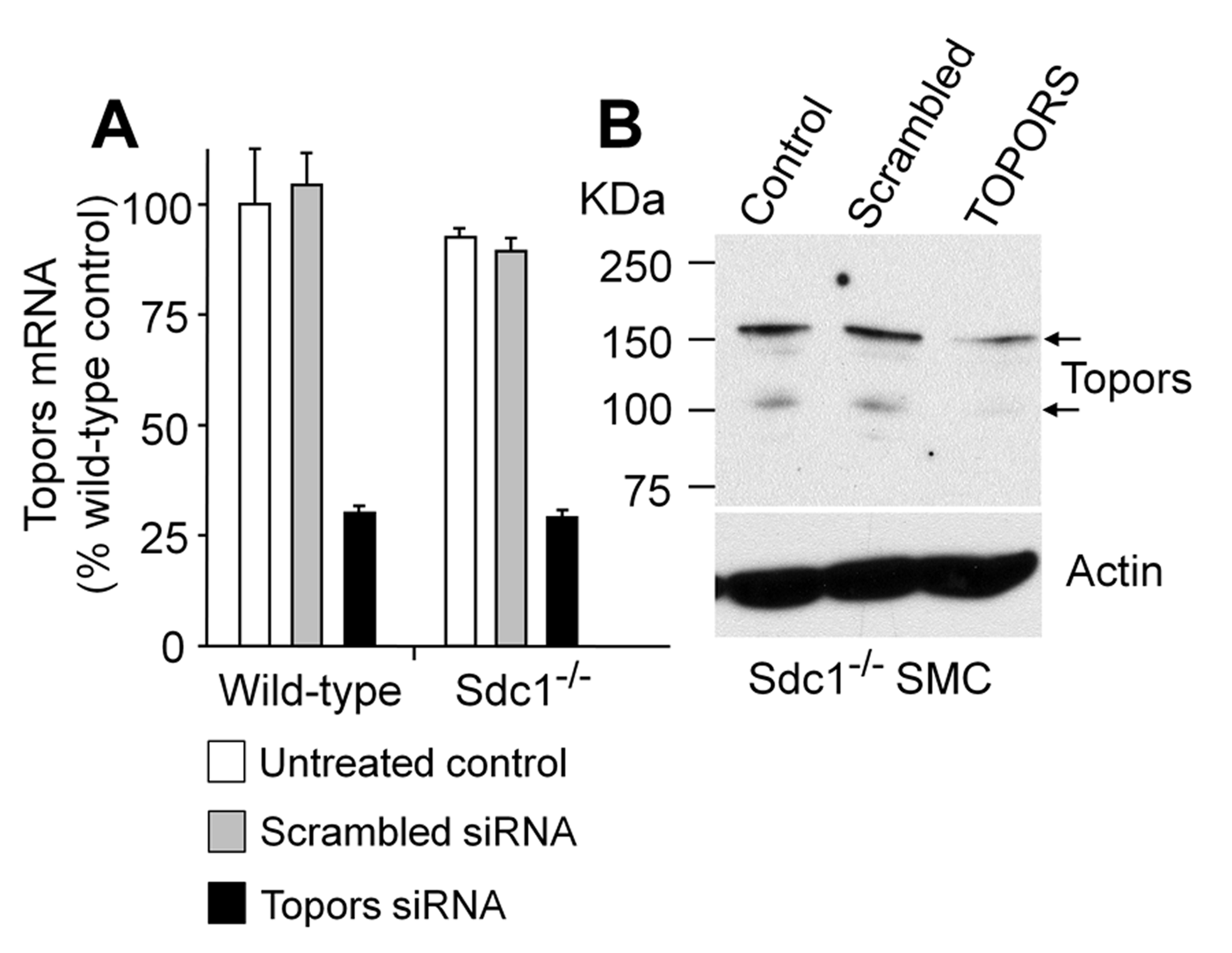

Supplement: Figure S1 — The effect of siRNA-mediated Topors knockdown on Topors mRNA and protein levels in SMCs. (B) Topors mRNA expression was determined by qPCR in samples from untreated control cultures and in cultures 48 hours after transfection with scrambled or Topors-targeted siRNA, as determined in a representative experiment with triplicate cultures. (C) Western blotting for Topors in total cell lysates from cultures of untreated Sdc-1 null cultures and cultures 48 hours after transfection with scrambled or Topors-targeted siRNA. Under these conditions Topors siRNA treatment resulted in a ∼70% Topors mRNA and protein knockdown. (TIF) [file pone.0043701.s001.tif]
